# Analysis of floodplain forest sensitivity to drought

**DOI:** 10.1098/rstb.2019.0518

**Published:** 2020-09-07

**Authors:** Natalia Kowalska, Ladislav Šigut, Marko Stojanović, Milan Fischer, Ina Kyselova, Marian Pavelka

**Affiliations:** 1 Department of Matter and Energy Fluxes, Global Change Research Institute of the Czech Academy of Sciences, Bělidla 986/4a, 60300 Brno, Czech Republic; 2 Department of Xylogenesis and Biomass Allocation, Global Change Research Institute of the Czech Academy of Sciences, Bělidla 986/4a, 60300 Brno, Czech Republic; 3 Department of Agrosystems and Bioclimatology, Faculty of Agronomy, Mendel University in Brno, Zemědělská 1, 61300 Brno, Czech Republic

**Keywords:** floodplain forest, drought, eddy covariance, radial stem variations, tree water deficit, gross primary production

## Abstract

Floodplain forests are very complex, productive ecosystems, capable of storing huge amounts of soil carbon. With the increasing occurrence of extreme events, they are today among the most threatened ecosystems. Our study's main goal was to assess the productivity of a floodplain forest located at Lanžhot in the Czech Republic from two perspectives: carbon uptake (using an eddy covariance method) and stem radius variations (using dendrometers). We aimed to determine which conditions allow for high ecosystem production and what role drought plays in reducing such production potential. Additionally, we were interested to determine the relative soil water content threshold indicating the onset and duration of this event. We hypothesized that summer drought in 2018 had the most significant negative effects on the overall annual carbon and water budgets. In contrast with our original hypothesis, we found that an exceptionally warm spring in 2018 caused a positive gross primary production (GPP) and evapotranspiration (ET) anomaly that consequently led in 2018 to the highest seasonal total GPP and ET from all of the investigated years (2015–2018). The results showed ring-porous species to be the most drought resistant. Relative soil water content threshold of approximately 0.45 was determined as indicating the onset of drought stress.

This article is part of the theme issue ‘Impacts of the 2018 severe drought and heatwave in Europe: from site to continental scale’.

## 1. Introduction

Floodplain forests are characterized as one of the world's most complex and dynamic ecosystems, with great habitat heterogeneity and diverse biota adapted to high spatial–temporal heterogeneity [1]. Also among the most biologically productive ecosystems, floodplain forests are able to store huge amounts of soil carbon [2] and their structure and production are closely tied to fluvial dynamics. The timing, depth and duration of flooding have been found to be the main determinants of structural complexity, species richness, species composition and primary productivity [3]. Even small changes in the relative contribution of individual water sources may drastically degrade the natural landscape and modify the hydrological regime [1]. Floodplain forests play a crucial role in carbon and nutrient cycles. Despite their importance as confirmed by the many roles they play in the environment, understanding is still lacking as to how variation in environmental conditions influences carbon-related floodplain ecosystem processes.

Ongoing climate change undeniably has a global impact on floodplain forests' decline. More frequently occurring extreme events associated with increased temperature and changed precipitation patterns rank floodplain forests among the most altered and threatened ecosystems [4]. Prolonged and intense drought alters hydrological regimes in these ecosystems. Increase in global air temperature by 3–4°C is predicted to eliminate 85% of all remaining wetlands [5]. With an anticipated increase in drought events’ frequency and intensity [6], it is extremely important to understand drought impact on ecosystems and to quantify the conditions limiting sustainable ecosystem functioning.

Drought is usually defined based on precipitation and air temperature using such standard climatological methods as the standardized precipitation–evapotranspiration index (SPEI) [7]. We emphasize, however, that the careful assessment of physiological variables is needed to determine the onset and intensity of drought stress in order to further evaluate the fate of forest ecosystems in the future climate.

Our study's main goal was to assess the productivity of a floodplain forest located at Lanžhot in the Czech Republic from two perspectives: carbon sequestration (using an eddy covariance method) and stem radial variation (measured using dendrometers). We focused especially upon finding which conditions allow for high ecosystem production and what role drought plays in reducing such production potential. In addition, we were interested to determine the thresholds of relative soil water content (rSWC) at which the ecosystem begins to exhibit signs of stress.

We hypothesize that summer drought in 2018 had the most significant negative effects on the overall annual carbon and water budgets during the study years (2015–2018) and that differences should be observed among the species oak (*Quercus robur L.*), hornbeam (*Carpinus betulus L.*) and ash (*Fraxinus angustifolia L.*) at the study site.

## 2. Material and methods

### (a) Site description

Lanžhot is a floodplain forest located 6.5 km north of the confluence of the Morava and Thaya rivers. The site's characteristics are presented in the electronic supplementary material, tables S1 and S2. Acosta *et al.* have described it in detail in [8]. Anthropogenic forest and hydrological management in the 1970s via building artificial dams in the study region intensified the drying of the study site. This also caused a loss of connectivity between the river and the forest. This consequently led to drier conditions by eliminating floods and decreasing mean annual amplitudes of water levels, which could have a significant effect on the remnant forest. Over the past 40 years, flooding at the study site occurred very rarely and only for short periods. The last flood there was in 2013, and the measurement site was flooded only in depressions located in the northern and eastern part of the footprint. The maximum level of water during this flood was 0.5 m.

Europe had seen a number of droughts in the twentieth century, but these have been more frequent in the past 20 years. In these two decades alone, Central Europe faced widespread drought events in 2003, 2015 and 2018 [9]. Because this region of South Moravia lays within the north of the Pannonian Region with its prevailing continental climate, it also was affected greatly by other drought events striking south-eastern Europe, including in 2007, 2012, and 2017.

### (b) Instrumentation and data processing

The most relevant instrumentation used is characterized in the electronic supplementary material, table S3. For each measured variable, both the instrument and its height (or depth) are given.

#### (i) Eddy covariance measurements

The eddy covariance fluxes were processed within a joint action organized by ICOS and the FLUXNET community in response to the 2018 drought [10]. Flux data from the 2015–2018 period followed the standard FLUXNET processing [11], including friction velocity (u*) filtering [12], and gross primary production (GPP) determination by night-time flux partitioning [13]. Latent heat fluxes were not corrected for energy balance closure.

Potential GPP was determined by the 95th quantile regression of GPP response to photosynthetically active radiation (PAR) during the 2016 growing season using non-rectangular hyperbola and predicting GPP values for the whole study period based on actual PAR measurements and using the obtained fit. Potential evapotranspiration (PET) was determined based on the Penman–Monteith equation, where the potential surface conductance (*G*_s_) was determined as the 95th quantile of the *G*_s_ to vapour pressure deficit (VPD) and *G*_s_ to global radiation response curves fitted using a modified Lohammar equation [14].

The computation of SPEI for the spring period April–June (three-month SPEI) and for the growing season period April–September (six-month SPEI) was made according to Beguería *et al.* [15] and Vicente-Serrano *et al.* [7] using an R-package SPEI [16]. The SPEI represents an anomaly in climatological water balance given by the difference between precipitation and reference evapotranspiration. The input data were obtained from the ERA5 climate reanalysis [17]. We used the period 1981–2010 as a baseline period for the SPEI computation.

#### (ii) Stem radius (dendrometers) measurements

Stem circumference variations were measured by automatic band dendrometers on six trees per species during the period 2016–2018. Owing to technical problems, the start of measurements in 2017 was postponed to 15 May and final growth was estimated based on inventory data and wood formation studies [18]. Resolution of the dendrometers was 1 µm and the measuring and storing interval was set to 10 min. Before installing dendrometers, the relatively thick outer bark of oaks and ashes was removed to reduce hygroscopic swelling and shrinkage of the bark tissues on stem radius records. The stem circumference data were converted into stem radius for further analysis. In addition to tree growth (irreversible changes), dendrometers also recorded tree water deficit-induced stem shrinkage (reversible changes), which was partitioned following Zweifel *et al.* [19,20]. We used the ‘zero growth line’ approach, which assumes that growth occurs only when the stem radius exceeds the previous maximum radius value [19,20]. The difference between the zero growth line and the current stem radius value is regarded as the relative measure of drought-related tree water deficit (TWD).

#### (iii) Relative soil water content computation

Soil water content was normalized in respect to lowest and highest values observed in the 2016–2018 period following Hartmann *et al.* [21] to produce rSWC. For this purpose, the weighted mean of SWC was computed from the SWC profile while considering soil layer thickness. Measurements affected by low temperatures (below +1.3°C) were removed and the gaps were filled using linear interpolation.

#### (iv) High-frequency leaf area index estimation

Light penetrating through the canopy was measured along transects produced by 25 PAR sensors. The records obtained were averaged along the whole transect in half-hourly resolution. Leaf area index (LAI) was optimized based on routines described for the SunSCAN (Delta-T Devices Ltd, Cambridge, UK). The resulting LAI values were accepted only when the sun was low above the horizon (zenith angles greater than 70° excluded) and cloudy conditions prevailed (records with clearness index greater than 0.4 excluded). Final LAI was produced by locally weighted smoothing (LOWESS) of filtered LAI estimates.

## 3. Results

### (a) Meteorological conditions and drought assessment

During the study years, the Lanžhot site was characterized by dry conditions in 2017 and 2018. The year 2016 could be considered as having conditions normal for the site. We therefore use 2016 as a reference, because the SPEI index indicated it to be the year with least drought stress (electronic supplementary material, table S5). Annual mean temperature was slightly lower in 2016 (10.9°C) compared to 2015 (11.5°C), 2017 (11.0°C) and 2018 (12.1°C). The year 2018 was characterized by the highest monthly mean air temperatures in April, May and August, and it reached the second-highest mean air temperatures among all the years for June and July (electronic supplementary material, table S4). Annual precipitation totals also differed among years, with similar values in 2016 and 2017 (504 and 505 mm, respectively) and substantially lower values of 425 mm in 2015 and 435 mm in 2018 (electronic supplementary material, table S4).

**Figure 1.**
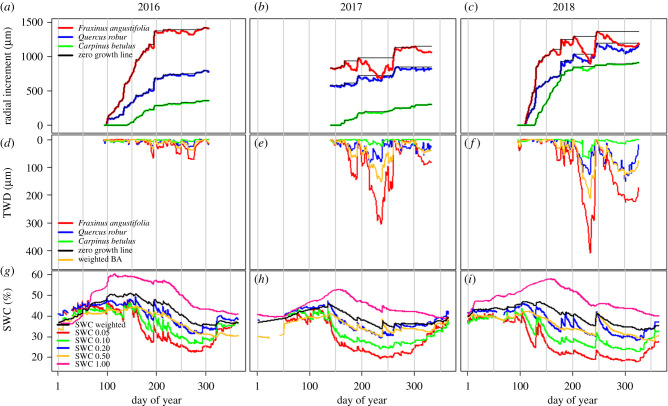
(*a*), (*b*), (*c*) Daily radial stem variations together with zero growth lines drawn in. (*d*), (*e*), (*f*) Daily tree water deficit (TWD). (*g*), (*h*), (*i*) Daily soil water content (SWC) in individual years. Colours denote species: *Fraxinus angustifolia L.* (red) *Quercus robur L.* (blue) and *Carpinus betulus L.* (green).

SWC distinctly differed among the studied years. The years 2017 and 2018 were significantly drier compared to 2016 at all measured depths (*p* < 0.05). In all years, SWC notably dropped in the second part of April, and in August of 2018, it decreased even below 20% (at 5 cm depth), whereas in 2016 and 2017, soil moisture always remained above this value (figure 1*g–i*). When comparing dry years 2017 and 2018, we observed significantly lower SWC values in 2018 in surface layers (5 and 10 cm depths) compared to 2017 (*p* < 0.05), while the 20 cm depth showed no differences. On the other hand, deeper soil layers (50 and 100 cm depth) were significantly moister in 2018 compared to 2017 (*p* < 0.001) (figure 1*h,i*). The SWC differences among years were probably caused by high evapotranspiration (ET) demand in the exceptionally hot 2017 and 2018 (electronic supplementary material, figure S6). On the other hand, more favourable distribution of precipitation (i.e. winter and May in 2018) might have caused the deeper soil layers to be refilled.

### (b) Intra-annual radial growth and tree water deficit

The contrasting weather conditions within the studied period (2016–2018) contributed to the observed differences in seasonal growth dynamics. Although the tree species showed similar growth patterns within the species and years, differences between growth rates by species and years were evident (figure 1*a*–*c*).

The year 2018, which, as mentioned, was characterized as overall the hottest with almost 70 mm lower annual sum of precipitation in comparison with 2016, presented the highest growth rates among all tree species (electronic supplementary material, table S4). Ash had the fastest growth rates, followed by oak, while hornbeam had the slowest in all three years. All species showed TWD increase starting in June in all three years (figure 1*d*–*f*). Nevertheless, TWD values were significantly higher in 2017 and 2018 compared to 2016 (*p* < 0.001). The increase in TWD followed the same pattern in all species and always coincided with the decrease in SWC (figure 1*g*–*i*). The highest TWD values were observed in ash trees, followed by oak, whereas hornbeam had lowest values in all three years. The weighted mean TWD (yellow line of figure 1*d*–*f*) was computed based on basal area distribution among individual tree species (electronic supplementary material, table S2).

### (c) Evapotranspiration, evaporative fraction and productivity of the ecosystem

**Figure 2.**
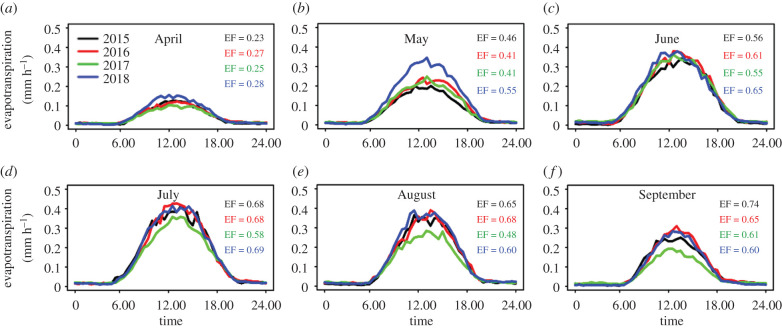
Monthly mean diurnal pattern of evapotranspiration for the growing season of each measured year. For each month of the year, mean evaporative fraction (EF (−)) was calculated. Evaporative fraction was defined as LE/(*H* + LE), where LE is latent heat flux and *H* is sensible heat flux.

During the growing season (April–September) of our flux measurement period (2015–2018), the year 2018 exhibited heightened ET in April and May compared with in previous years. Interesting results were also observed in 2017, when ET was the lowest in April, July, August and September in comparison with other measured years (figure 2).

**Figure 3.**
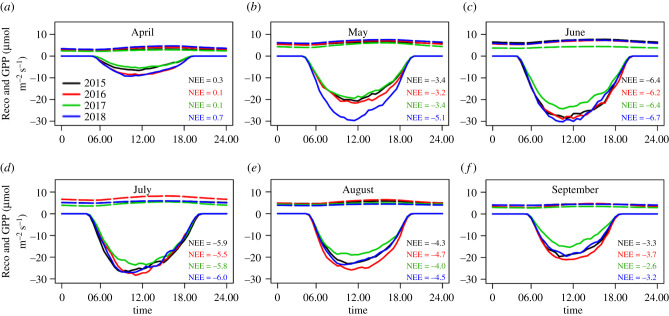
Monthly mean diurnal pattern of ecosystem respiration (reco; broken lines) and gross primary production (GPP; solid lines) for growing season of each measured year. Mean net ecosystem exchange (NEE) (μmol m^−2^ s^−1^) was calculated for each month of the year.

**Figure 4.**
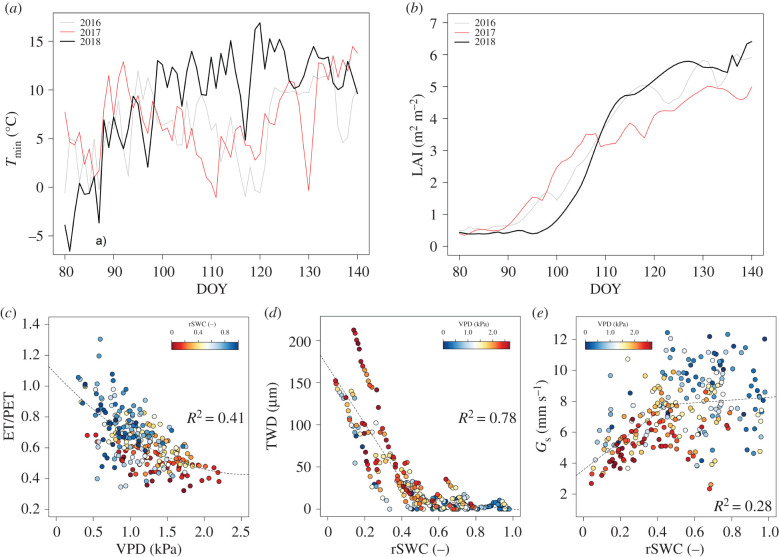
(*a*) Daily courses of minimum air temperature (*T*_min_) and (*b*) daily courses of leaf area index (LAI) for the measured years. Relationships between (*c*) evapotranspiration/potential evapotranspiration (ET/PET) and vapour pressure deficit (VPD), (*d*) tree water deficit (TWD) and relative soil water content (rSWC), (*e*) surface conductance (*G*_s_) and rSWC for days of year (DOY) 130–250 of 2016–2018.

Substantial increase in GPP was observed during 2018 already in May. This was connected with earlier development of leaves during the warmer spring in 2018. In all previous years, enhanced vegetation activity started in June. The year 2017 was characterized by delayed leaf development and spring frost (figures 3 and 4*a*,*b*; electronic supplementary material, figure S3).

The relationships between different physiological variables were also analysed to determine the onset and intensity of drought stress (figure 4*a*–*e*). Relationships were calculated using second-order polynomial fitting (figure 4*c*; electronic supplementary material, figures S8–S13) and segmented regression (figure 4*d*,*e*; electronic supplementary material, figure S14). The best goodness of fit was observed where TWD significantly declined with increased rSWC until rSWC reached about 0.45 (figure 4*d*). This figure presents an rSWC threshold where TWD becomes insensitive to rSWC. In addition, under the highest rSWC, there was already no observed decrease in TWD (figure 4*d*). In all cases, VPD and rSWC showed a strong negative correlation (figure 4*c*–*e*; electronic supplementary material, figures S9, S11–S14).

## 4. Discussion

Our initial hypothesis that the summer drought in 2018 had the most significant negative effect on the overall annual carbon and water budgets during the study years was not supported. We found that the positive spring anomaly overcompensated the reduced water availability owing to summer drought.

The significantly lower evaporative fraction in July, August and September in 2017 (figure 2; electronic supplementary material, table S4, figure S5) in comparison with other years owing to reduced SWC in this year (figure 1; electronic supplementary material, table S4) may reflect the forest's losing its cooling characteristics. Cooling that is needed but not provided may consequently lead to temperature increase which, in combination with already existing water stress owing to reduced SWC, may extend the scale of stress caused by heat [22]. At the same time, a decline in GPP (figure 3) as well as in normalized GPP (electronic supplementary material, figure S4) indicates reduced atmospheric CO_2_ uptake. Reichstein *et al.* [23] have shown that summer drought reduces or even inhibits photosynthesis and as a consequence can lead to reduced carbon uptake by the ecosystem. Both cooling effect and sequestration capability are oft-discussed attributes of wetland forest in relation to climate change mitigation potential [24]. Owing to human activities that lead to significant alterations in floodplain forest, however, the potential today for these ecosystems to mitigate climate change has become limited. In addition, the more frequent occurrence of extreme events causes vegetation stress, which, according to Middleton & Souter [25], is a primary indicator of unacceptable levels of wetland alteration. Middleton & Souter explain that early detection of stress might be used to signal a need for hydrologic remediation, thereby allowing action to be taken to reduce tree stress and ultimately to mitigate future climate change problems [25]. Moreover, a developed forest's health assessment that signals remediation could be a key point for reviving forested wetlands suffering from drought.

Our study demonstrates that the warm spring of 2018 caused positive GPP and ET anomalies at the site (figures 2, 3 and 4*a*) that outweighed the small reduction of these two processes in the later part of the season. Flach *et al.* [26] also observed that temperature anomalies in spring and summer lead to GPP anomalies. Consequently, in our study, 2018 had the highest GPP and ET seasonal total among all years (electronic supplementary material, figures S1; figures 2 and 3; electronic supplementary material, table S4), which was in contrast with our original hypothesis. We can find examples in the literature of increased air temperature in spring causing earlier vegetation activity [27,28]. On the one hand, warmer springs with possibly sufficient water and nutrients availability (owing to decomposition of autumn leaf fall) can result in positive GPP response; on the other hand, earlier vegetation activity can lead to soil moisture depletion and ultimately decrease productivity in summer, causing drought [27]. In our study, however, we did not observe the second effect but assume we might see it in future if sufficient refilling is not achieved during winter.

In addition to the evidence that a drought event can have a significant influence on the ecosystem, another important aspect of this study is its justification for careful assessment of physiological variables during a drought event that is crucial in the context of the forest ecosystem's fate in the future climate. During stress conditions caused by increased VPD in the environment, it is possible to expect that higher VPD will lead to increased ET [4]. Further increase in VPD may drive an increase in ET, because in this case, increased atmospheric demand exceeds plant response to conserve water [29]. Nevertheless, owing to the evolution of plants, stomata to regulate the exchange of water and carbon in the most optimal way, in a forest with rising VPD, there is more often observed ET decrease instead of increase [28,30].

In contrast with all other analysed years, 2017 was characterized by systematically lower LAI as well as ET and GPP. We suspect that a possible explanation for this might relate to the spring frost in 2017, which negatively affected LAI development such that, as a consequence, the ecosystem was not able to compensate for this and thus the whole year sum of GPP and ET was affected (figures 3 and 4*a*,*b*; electronic supplementary material, figure S3). Similar results have been presented by Kramer *et al.* [31]. This may further suggest that the forest was already unable to overcome this stress because additional stress represented by low SWC appeared (figure 1*h*; electronic supplementary material, S3; figure 4*a*). Similar dependencies between frost and LAI were observed in 2016, but, because that year was not limited by water, this negative effect of air temperature could be overcome by later canopy development.

Our results show differences among species. Ash, despite clear diminished radial increment during August 2017 and 2018 owing to decreased rSWC at that time (electronic supplementary material, figure S2), was characterized by the highest productivity over the measurement period as a whole. This might be explained by higher photosynthetic nitrogen-use efficiency in ash, as stated by Kazda *et al.* [32], whose study was conducted in close vicinity to our study site. Nevertheless, social position within the stand may explain hierarchy within the tree species [32]. Ash and oak are in dominant and co-dominant tree strata, while hornbeam is situated in the understorey (electronic supplementary material, table S2). Hence differences in availability of photosynthetic active radiation can explain the faster growth rates of ash and oak. Different behaviour may also be explained by differences in xylem anatomy, which in turn influences species-specific growth phenology [33]. Ash and oak are ring-porous tree species and were characterized by earlier growth onset. Meanwhile hornbeam, as a diffuse-porous species, was characterized by a consistent late growth onset (figure 1*a*–*c*). Earlier growth onset of oak and ash is probably owing to restoration of the water-conducting system after embolism of previous years' large vessels, and this happens before leaf budburst [18]. Hence, ash and oak had comparatively longer growth duration as compared to hornbeam (figure 1*a*–*c*). On the other hand, an earlier and more vigorous start of the growing season during the warm spring 2018 caused a significant increase in growth of all investigated species (figures 1*a*–*c*, 3, 4*a*). Our study revealed large differences in values but a very similar pattern of TWD in the studied tree species. Evidently, an increase in TWD is attributable to decreasing water potentials caused by imbalances between transpiration and root water uptake [20]. Therefore, similar patterns of TWDs among the studied tree species (figure 1*d*–*f*) can be explained by the changes in tree water status (reversible stem changes) [33]. The noted differences in values of TWD among tree species are most probably linked to species-specific water use, but different anatomies of xylem and bark tissues also can contribute significantly to stem radial variations [34]. Further study to investigate species-specific water use and xylem architecture might explain the different behaviour of the different tree species. Nevertheless, our study found TWD to be a very reliable and sensitive indicator of the occurrence of stress.

Finally, a special interest in our study was to determine an rSWC threshold that would indicate the onset and duration of drought events as sensed by the ecosystem rather than being determined as a climatological anomaly. Analysis of *G*_s_, ET/PET and TWD response to rSWC allowed setting such rSWC threshold to approximately 0.45, where a relatively clear change in the ecosystem responses occurred (figure 4*d*,*e*; electronic supplementary material, figures S7, S8, S10).

## 5. Conclusion

In characterizing the productivity of floodplain forest, we found that the warm spring in 2018 caused a positive GPP and ET anomaly that outweighed the negative effect of later summer drought. As a consequence, 2018 had the highest GPP and ET seasonal total among all the investigated years. The current observed behaviour is probably dependent on sufficient soil moisture refilling, the future extent of which is questionable in a warming climate, as we can also document by our SWC data.

In our study, there were visible differences between the species in response to the dry conditions. Ring-porous species seemed to be more drought resistant owing to their efficient conductive system, but also with earlier growth onset they could profit from warmer and wetter spring conditions. From the three species investigated, and despite a clear decrease in radial increment in August 2017 and 2018 owing to decrease in rSWC, *Fraxinus angustifolia L.* was the most productive species.

Increase in TWD showed, however, the same pattern in all three species and always was associated with a decrease in SWC. Relative SWC threshold of approximately 0.45 was determined by several independent methods to indicate the onset of drought stress.

## Supplementary Material

Data supporting study presented as a tables and graphs

## Data accessibility

Data used in our publication can be used. All articles referring to them are provided in the References section in the main manuscript.
