# Supplementary material for: Analysis of floodplain forest sensitivity to drought
Source: Philos Trans R Soc Lond B Biol Sci. 2020 Sep 7;375(1810):20190518. doi: 10.1098/rstb.2019.0518 (PMC7485104; doi:10.1098/rstb.2019.0518)
Supplement: Data supporting study presented as a tables and graphs [file rstb20190518supp1.pdf]

Supplementary material

Table 1S. Characteristics of the studied site.

Site name	Lanžhot
Location	South Moravian Region of Czech Republic
Latitude	48.6815483 N
Longitude	16.9463317 E
Elevation (m a.s.l.)	150
Topography	floodplain
Ecosystem type	Mixed deciduous-broadleaf forest
Main species	English oak, narrow-leaved ash, hornbeam
Mean canopy height (m)	27
Soil type	Eutric Humic Fluvisol, Haplic Fluvisol, and Eutric Fluvisol (according to the FAO 2014 Classification)
Minimal soil depth (cm)	60
Long-term mean temperature (°C)	9.5
Stand age (years)	110
Long-term mean annual precipitation (mm)	517
Mean ground water level (m)	-2.7

Table 2S. Stand structural characteristics: diameter at breast height (DBH), mean tree heights (Height), basal area (BA), share of the stand basal area (Share in BA_{stand}).

Tree species	No. tree ha^{-1}	DBH (cm)	Height (m)	BA species (m^2 ha^{-1})	Share in BA_{stand} (%)
Quercus robur	32	51.8 ± 12.3	31.0 ± 3.6	7.13	23.5
Fraxinus angustifolia	48	56.4 ± 9.2	35.9 ± 3.4	12.30	40.6
Ulmus laevis	6	38.9 ± 2.2	28.8 ± 1.8	0.35	1.2
Carpinus betulus	148	27.7 ± 11.2	23.4 ± 6.9	10.41	34.4
Acer campestre	4	19.8 ± 7.0	20.0 ± 0.6	0.07	0.2
Tilia cordata	2	18.3	11.8	0.03	0.1

Mean values ± standard deviations are shown.

Table 3S. Instrumentation of the studied site.

Site name: Lanžhot	Sensor	Height/depth (m)
EC	LI-7200RS, LI-COR, Inc., NE, USA<br>HS-50, Gill Instruments, UK	44
Radiation	CNR 1, Kipp & Zonen, NL	44
Air temperature	EMS33, EMS Brno, CZ	36
Relative humidity	EMS33, EMS Brno, CZ	36
Wind speed	Wind Sonic 2D, Gill Instruments, UK	36
Precipitation	386C, MetOne, OR, USA	44
Soil temperature	Pt100, Sensit, CZ	-0.05; -0.10; -0.20;<br>-0.50; -1
Soil water content (SWC)	CS616L, Campbell Scientific, Inc., UT, USA	-0.05; -0.10; -0.20;<br>-0.50; 1
Stem increment measurements	Dendrometer Increment sensor DRL 26C, EMS Brno, CZ	1.8-2
Radiation (transect from 25 PAR sensors)	EMS12, EMS Brno, CZ	1
Radiation (PAR)	LI-190/R, Quantum sensor, LI-COR, USA	42

Table 4S. Monthly means of air temperature (Ta), Gross Primary Production (GPP), Ecosystem Respiration (Reco), Global Radiation (Rg), Vapor Pressure Deficit (VPD), Evaporative Fraction (EF), and monthly totals of Precipitation (P), Evapotranspiration(ET) and Potential Evapotranspiration (PET).

Year		Ta [°C]	P [mm month^{-1}]	ET [mm month^{-1}]	PET [mm month^{-1}]	GPP [μmol m^{-2} s^{-1}]	Reco [μmol m^{-2} s^{-1}]	Rg [W m^{-2}]	VPD [hPa]	EF [-]
2015	Jan	2.4	27.0	4.72	25.03	0.1	0.8	33.7	1.5	0.14
	Feb	2.1	22.4	4.87	26.90	0.0	0.6	75.6	1.7	0.15
	Mar	6.5	33.0	15.39	59.23	0.2	1.2	121.1	3.6	0.14
	Apr	10.8	35.8	33.22	107.35	2.5	2.7	209.2	6.6	0.23
	May	14.9	23.6	56.13	101.13	8.7	5.9	195.9	6.0	0.46
	Jun	19.1	16.8	91.03	144.50	12.9	6.9	273.3	9.2	0.56
	Jul	23.1	33.2	115.23	189.65	12.0	5.4	257.8	14.1	0.68
	Aug	23.3	93.6	94.06	168.08	9.4	5.2	222.7	13.6	0.65
	Sep	15.9	36.8	62.43	86.52	7.0	4.3	154.6	6.1	0.74
	Oct	9.8	52.0	21.31	36.17	2.5	3.0	69.4	2.3	0.54
	Nov	7.5	34.6	9.93	38.50	0.1	1.5	53.4	2.8	0.21
	Dec	3.3	15.8	5.00	11.10	0.0	1.2	25.8	0.8	0.11
2016	Jan	-0.3	19.2	6.74	15.98	0.1	0.8	37.6	0.9	0.15
	Feb	5.9	53.2	12.98	30.40	0.0	1.2	59.3	2.2	0.21
	Mar	6.1	17.0	15.78	50.26	0.4	1.7	105.3	2.9	0.15
	Apr	10.8	49.2	34.44	79.98	3.5	3.4	179.9	5.2	0.27
	May	15.6	62.6	68.73	110.72	9.5	6.1	230.3	6.3	0.41
	Jun	19.8	46.6	101.76	131.20	13.2	6.3	265.6	8.2	0.61
	Jul	21.4	114.0	115.42	141.28	11.7	7.2	253.7	9.1	0.68
	Aug	19.4	40.6	96.71	113.75	11.0	5.4	217.2	7.5	0.68
	Sep	18.3	20.4	68.81	99.30	7.8	3.9	173.6	7.5	0.65
	Oct	9.2	44.4	22.83	28.43	2.3	2.9	70.0	1.9	0.46
	Nov	4.7	28.2	7.13	20.78	0.1	1.7	47.4	1.4	0.14
	Dec	0.3	8.6	3.76	15.99	0.1	1.0	33.1	1.1	0.11
2017	Jan	-4.6	11.6	2.78	19.20	0.1	0.9	49.0	1.1	0.09
	Feb	2.6	19.6	5.79	30.37	0.0	0.8	64.6	2.0	0.12
	Mar	8.9	15.0	14.53	78.93	0.1	1.6	133.0	5.0	0.12
	Apr	9.7	62.0	30.03	79.04	2.1	2.5	156.7	5.1	0.25
	May	16.1	35.8	65.96	132.52	8.3	5.0	241.6	8.0	0.41
	Jun	21.3	27.4	99.89	186.13	10.6	4.0	282.0	13.6	0.55
	Jul	21.4	62.6	98.87	165.49	10.4	4.5	249.2	11.4	0.58
	Aug	22.1	74.2	77.22	167.16	8.1	4.5	223.3	12.3	0.48
	Sep	14.5	74.0	47.34	65.51	5.1	3.1	123.2	4.4	0.61
	Oct	11.5	48.8	25.82	48.64	1.9	2.4	84.6	3.3	0.39
	Nov	5.6	34.6	8.31	27.38	0.0	1.6	43.1	1.8	0.22
	Dec	2.4	39.4	6.03	19.85	0.1	1.0	31.5	1.5	-
2018	Jan	3.1	39.6	5.69	18.37	0.1	1.0	30.3	1.2	0.22
	Feb	-1.4	19.8	6.16	22.13	0.3	1.4	68.7	1.2	0.14
	Mar	3.2	13.0	9.07	46.32	0.1	1.1	104.1	2.6	0.10
	Apr	15.8	26.2	40.70	131.06	3.5	3.9	207.3	8.7	0.28
	May	18.4	56.4	92.32	132.39	12.5	6.8	241.4	7.9	0.55
	Jun	20.4	32.4	102.48	144.57	13.2	6.5	241.5	9.5	0.65
	Jul	21.8	64.2	116.38	167.17	11.6	5.4	252.1	11.7	0.69
	Aug	23.5	24.6	102.99	191.75	9.6	4.1	237.3	14.2	0.60
	Sep	17.3	101.4	63.21	97.91	6.7	4.2	170.4	7.0	0.60
	Oct	13.3	5.4	29.52	78.85	3.4	3.1	104.0	5.3	0.54
	Nov	6.8	16.2	9.82	32.15	1.4	3.1	43.7	2.4	-
	Dec	2.2	35.5	7.99	19.70	0.1	1.4	27.6	1.7	0.06

Table 5S. The standardized precipitation-evapotranspiration index (SPEI) index computed over 6 months and 3 months time scale for individual years.

SPEI	2015	2016	2017	2018
3 months interval (April-June)	-1.69	-0.35	-1.84	-1.49
6 months interval (April-September)	-1.60	-0.25	-1.15	-1.41

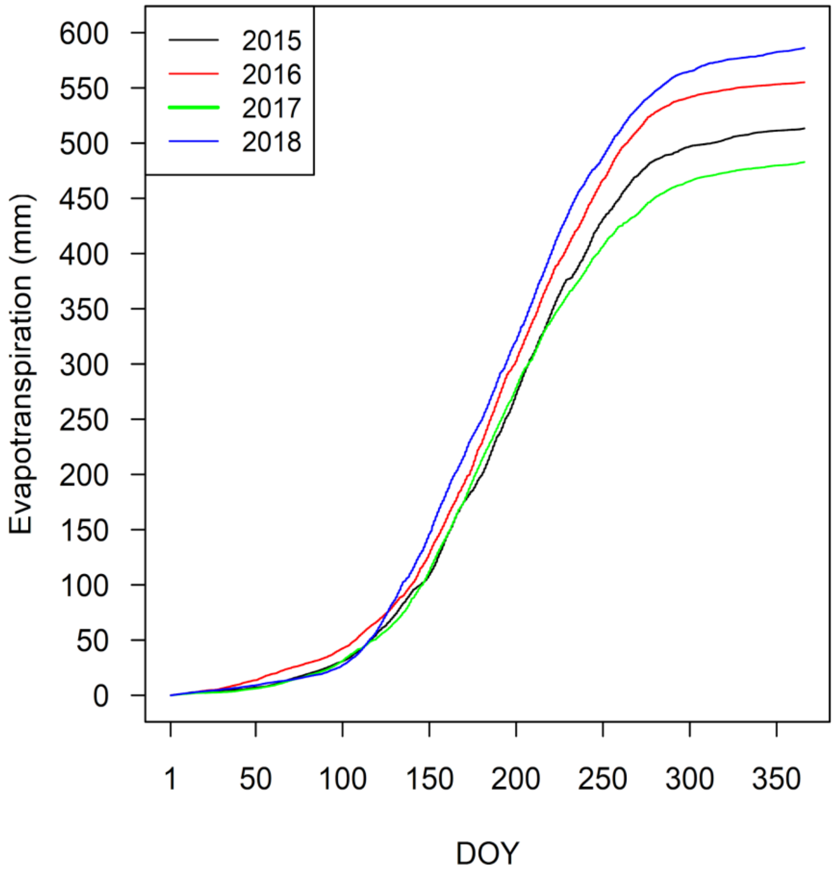

Figure 1S. Cumulative sum of evapotranspiration for individual years 2015-2018.

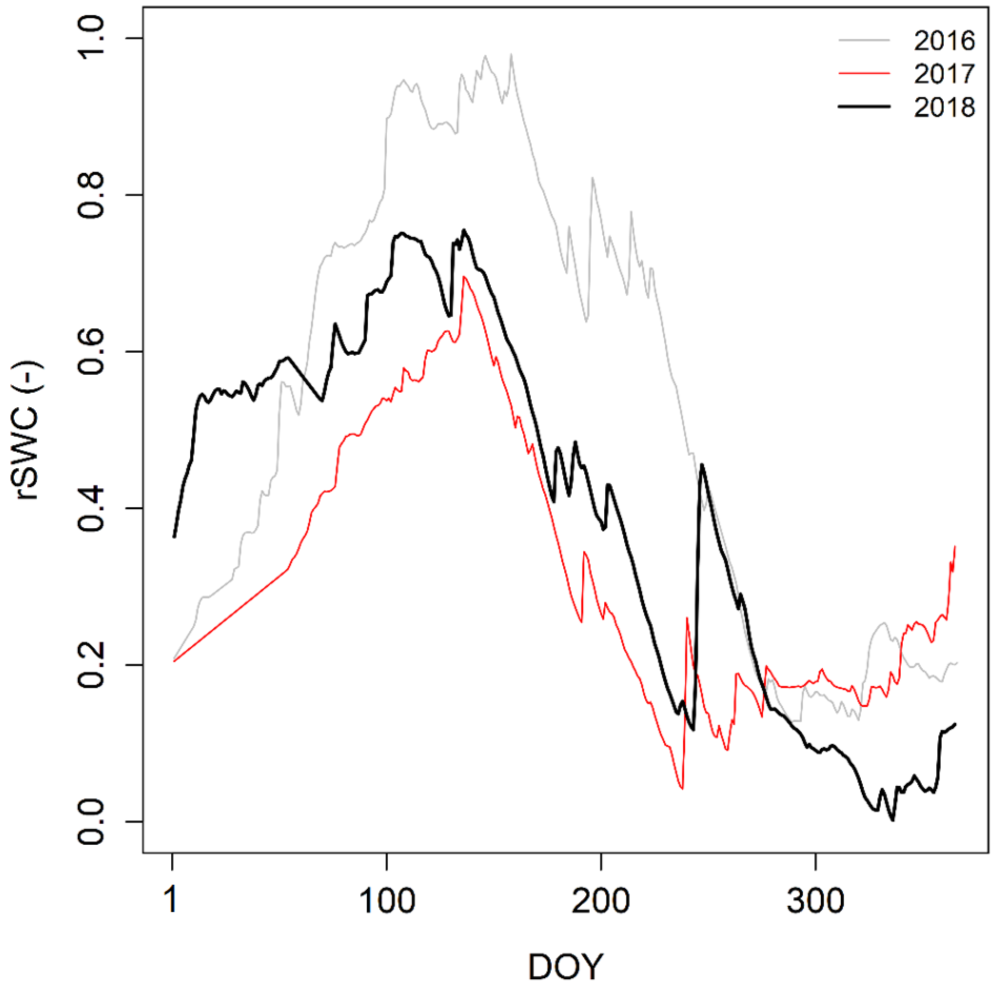

Figure 2S. Relative Soil Water Content (rSWC) time series for individual years 2016-2018.

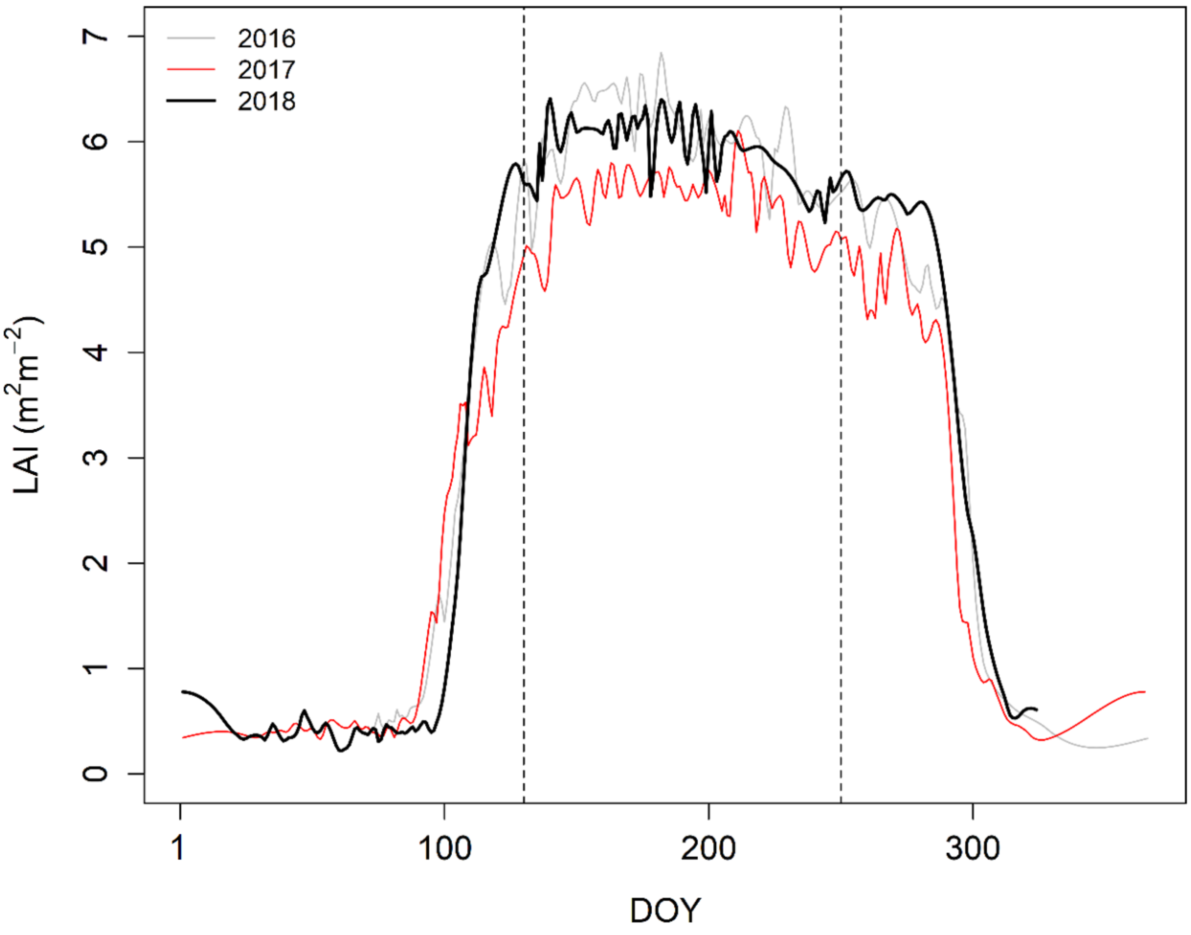

Figure 3S. Leaf Area Index (LAI) time series for the period 2016-2018. Vertical lines indicate the limits of the growing season.

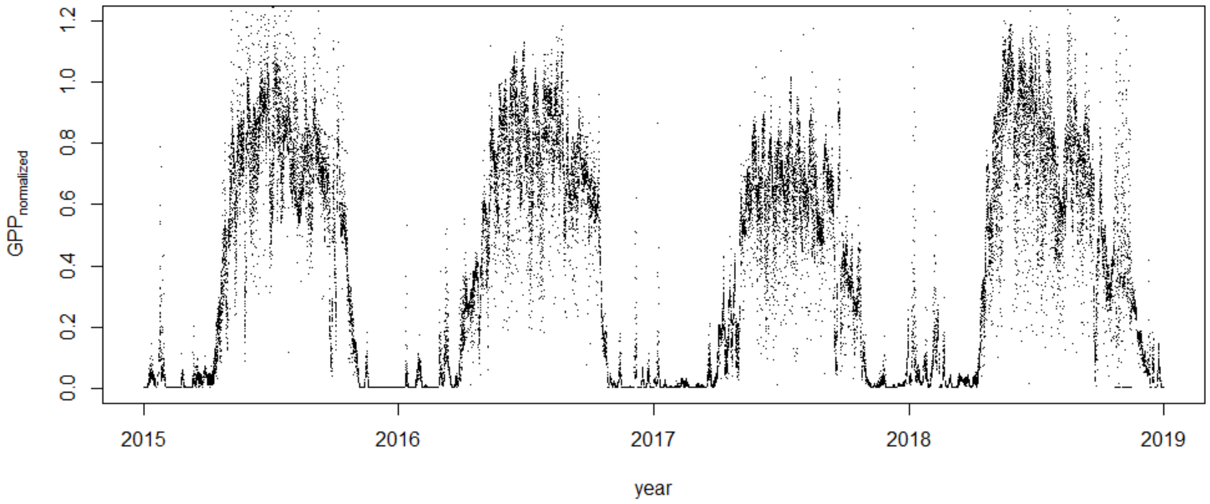

Figure 4S. Normalized gross primary production (GPP) time series for the period 2015-2018.

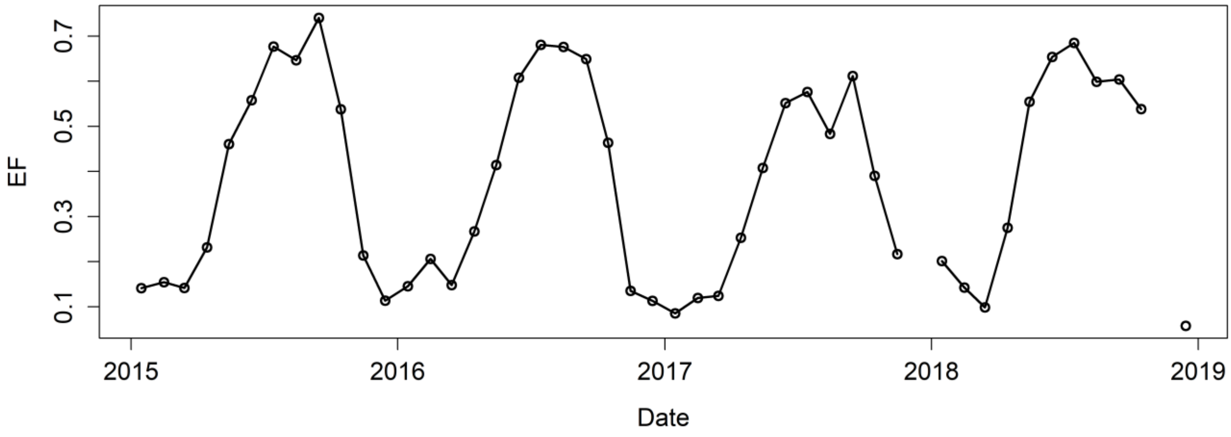

Figure 5S. Monthly means of the evaporative fraction (EF) for the period 2015-2018.

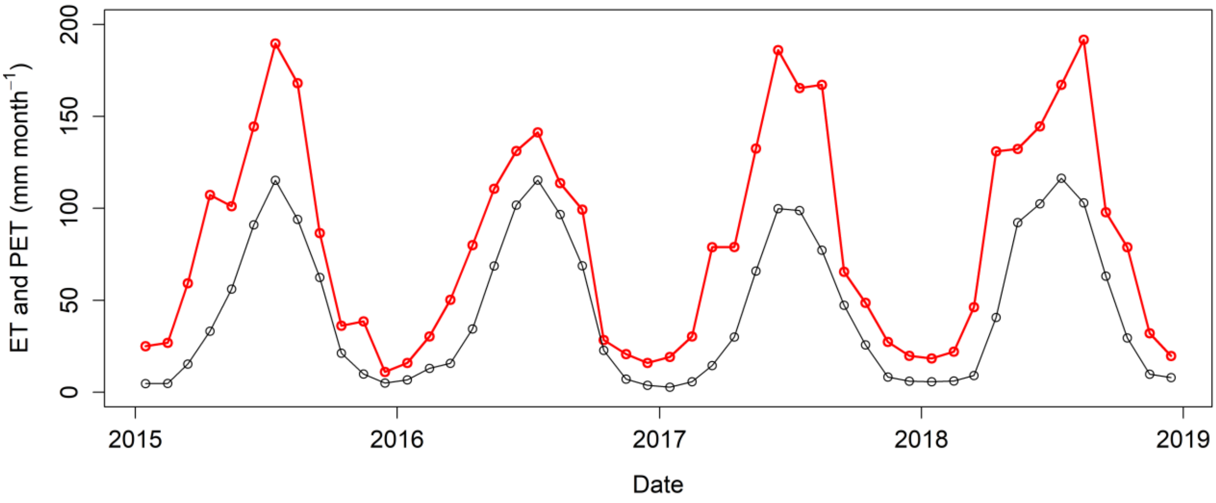

Figure 6S. Monthly means of evapotranspiration (ET; black line) and potential evapotranspiration (PET; red line) for the period 2015-2018.

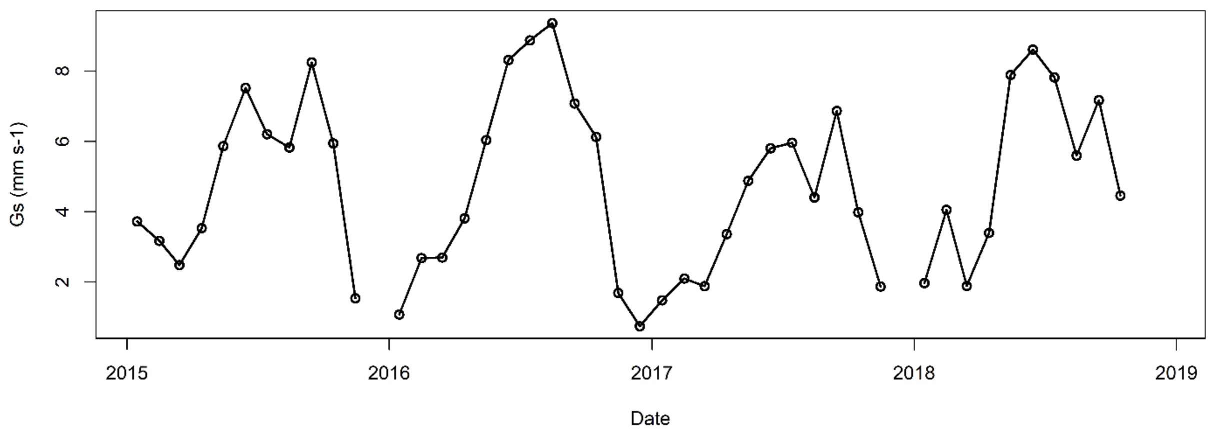

Figure 7S. Monthly means of surface conductance (G_s) for the period 2015-2018.

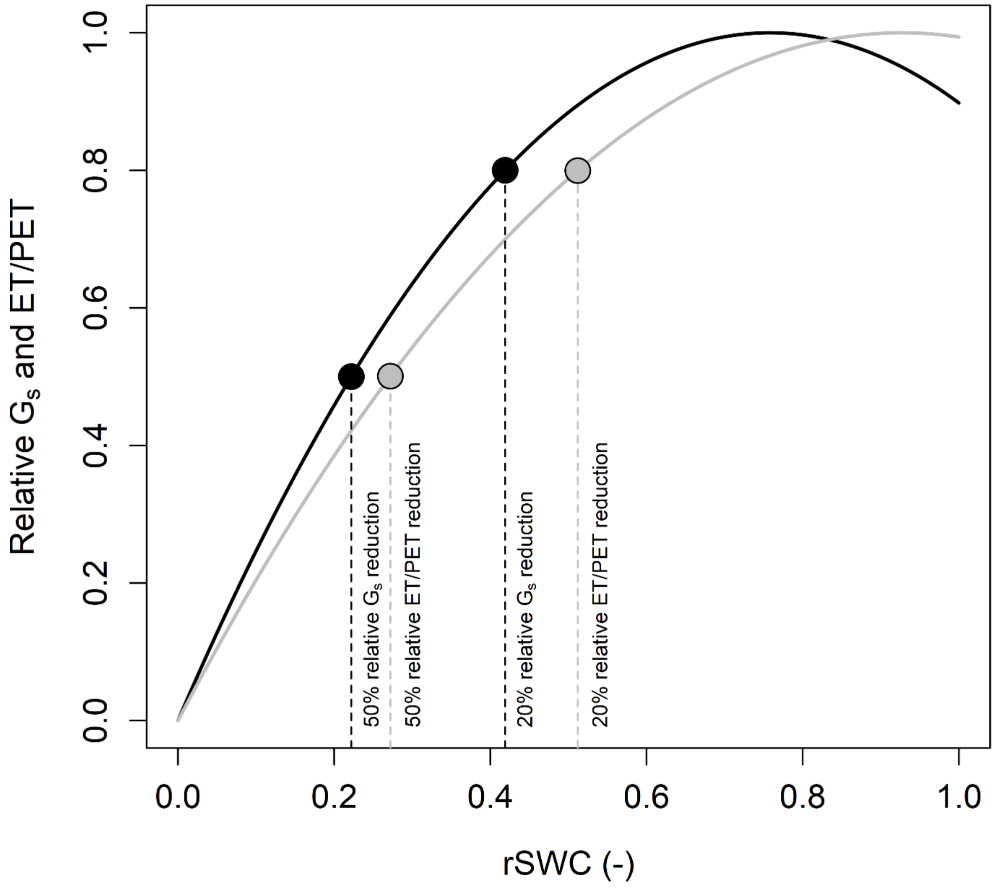

Figure 8S. Determination of rSWC thresholds for given reduction percentage of selected variables using the polynomial functions. Surface conductance (G_s) and evapotranspiration to potential evapotranspiration ratio (ET/PET).

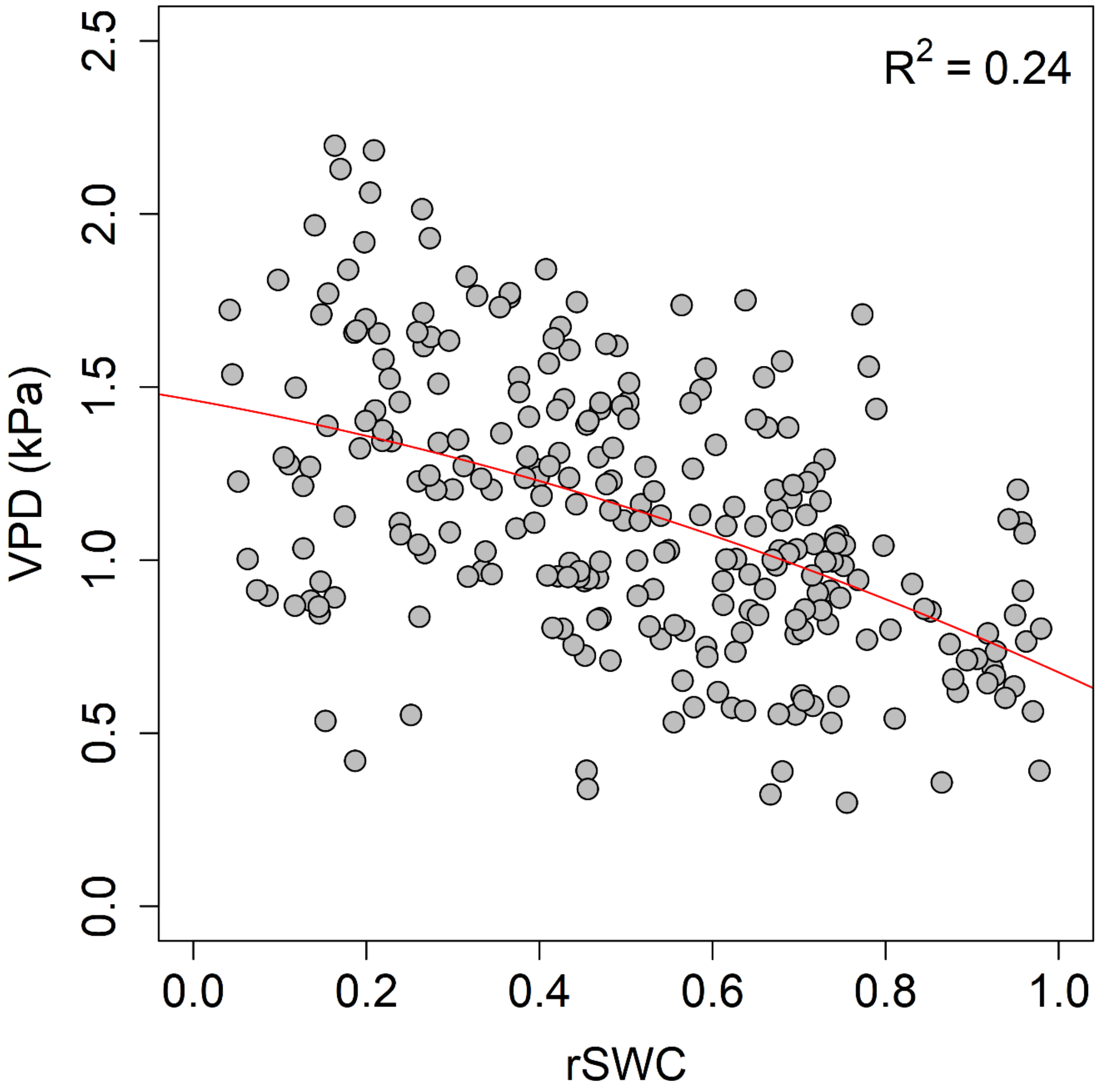

Figure 9S. Relationships between vapor pressure deficit (VPD) and relative soil water content (rSWC) for days of year 130–250 of 2016–2018. Relationships were calculated using the second-order polynomial fitting (red line), (p<0.05).

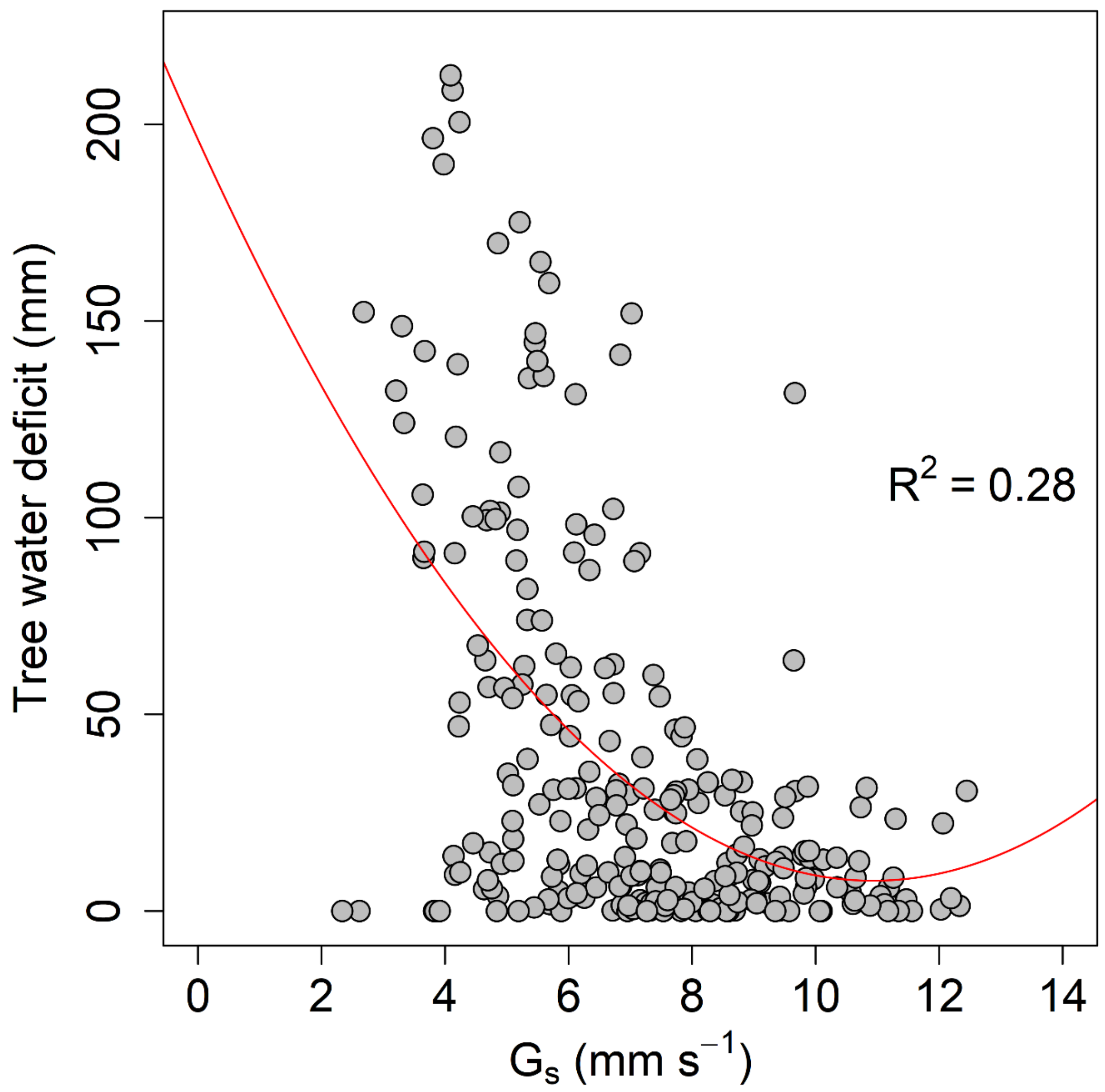

Figure 10S. Relationships between tree water deficit (TWD) and surface conductance (G_s) for days of year 130–250 of 2016–2018. Relationships were calculated using the second-order polynomial fitting (red line), (p<0.05).

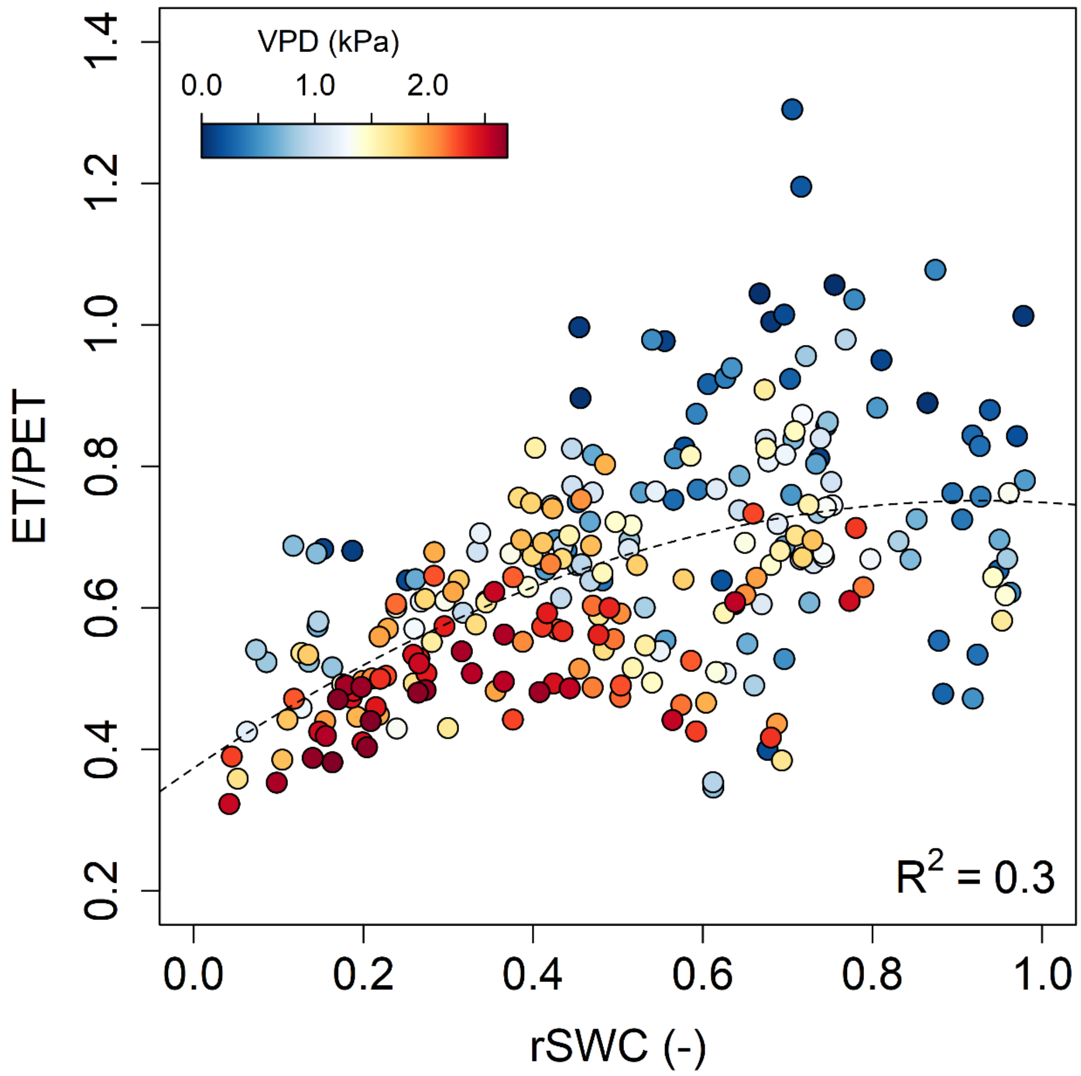

Figure 11S. Relationships between ET/PET and rSWC for days of year 130–250 of 2016–2018. Relationships were calculated using the second-order polynomial fitting (dashed line), (p<0.05).

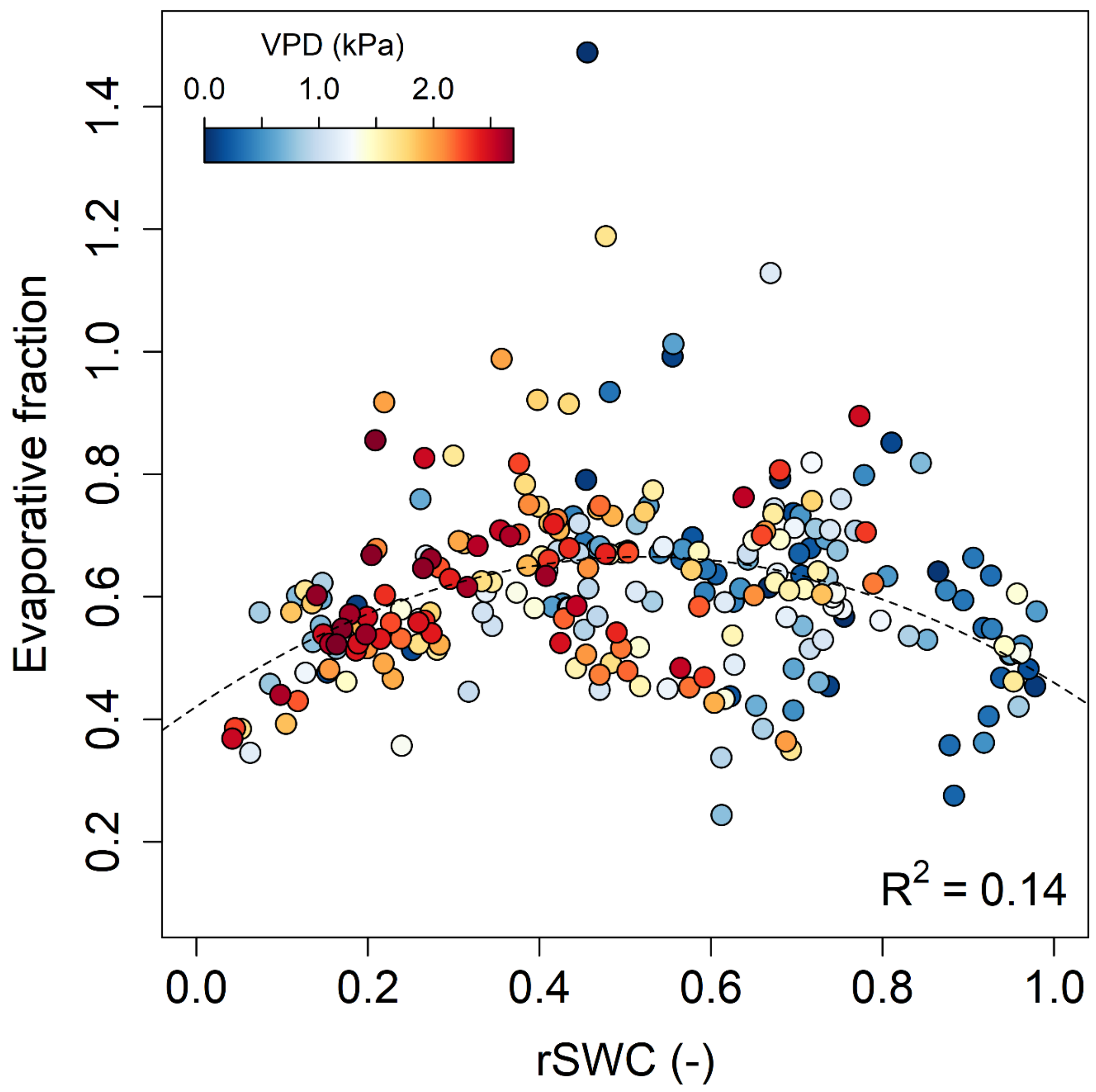

Figure 12S. Relationships between EF and rSWC for days of year 130–250 of 2016–2018. Relationships were calculated using the second-order polynomial fitting (dashed line), (p<0.05).

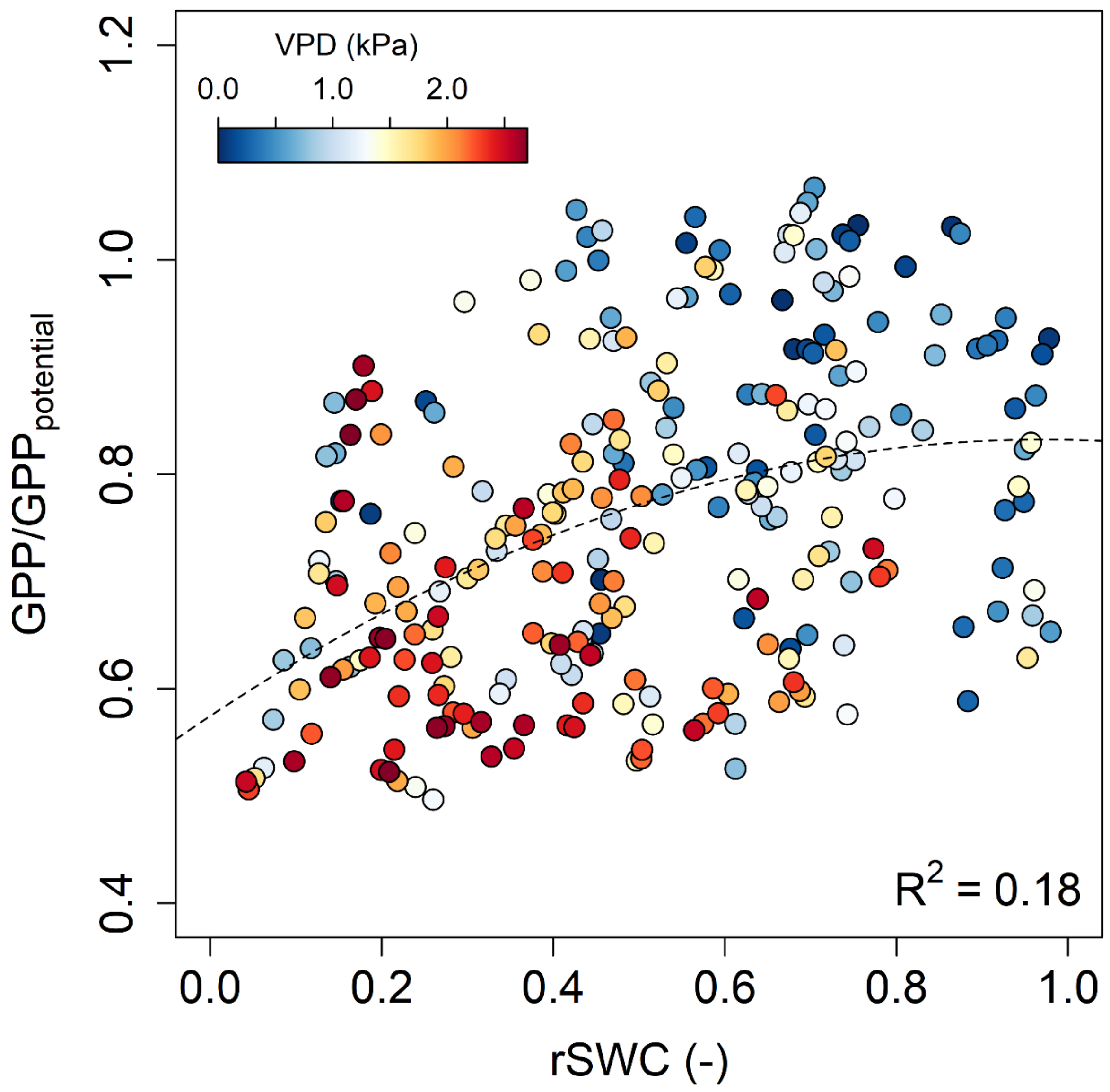

Figure 13S. Relationships between GPP/GPPpotential and rSWC for days of year 130–250 of 2016–2018. Relationships were calculated using the second-order polynomial fitting (dashed line), (*p*<0.05).

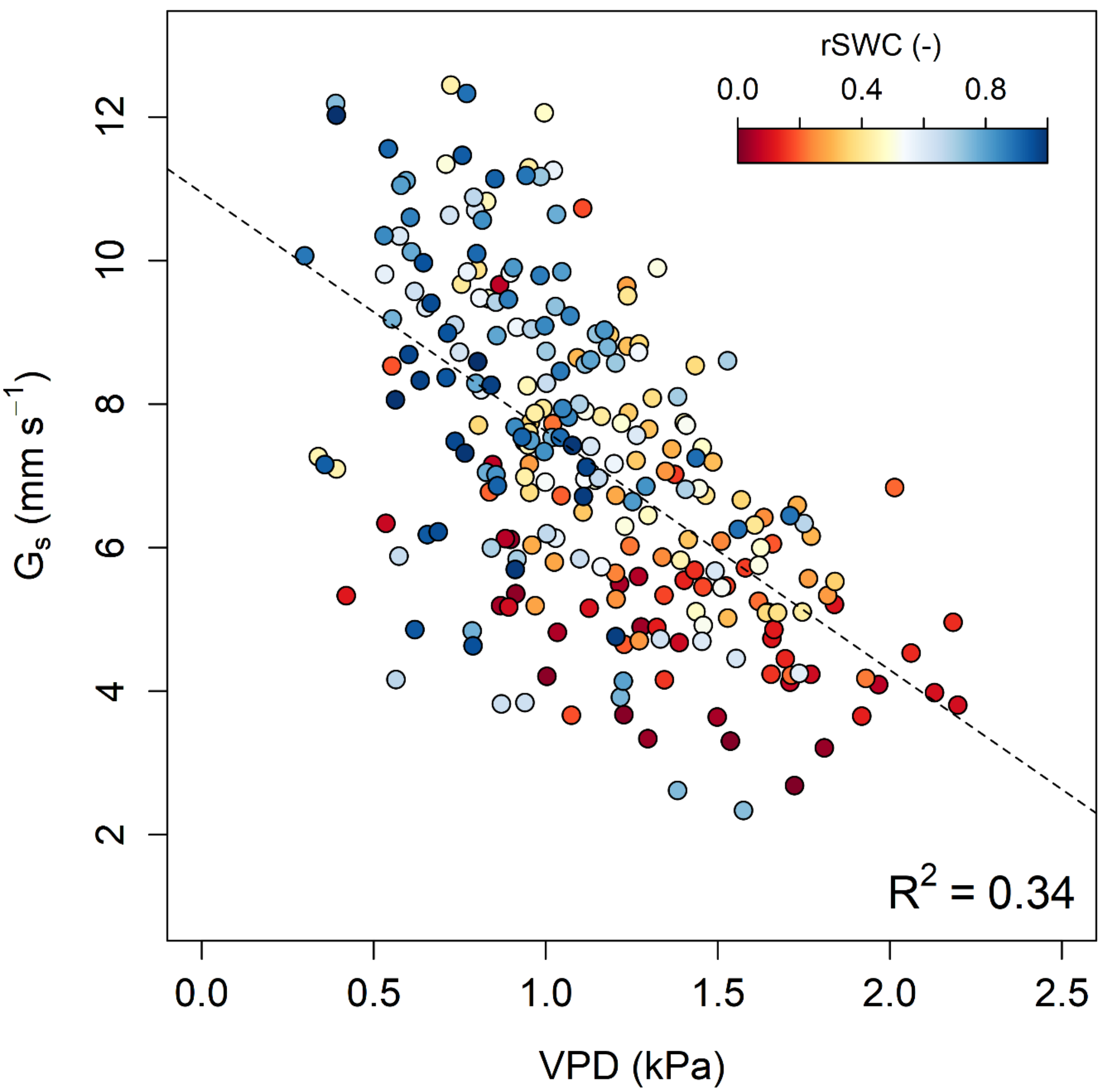

Figure 14S. Relationships between G_s and VPD for days of year 130–250 of 2016–2018. Relationships were calculated using segmented regression (dashed line), (p<0.05).
